# Behind the billions: policies, politics and power of the Global Financing Facility for women’s, children’s, and adolescents’ health

**DOI:** 10.1080/16549716.2025.2554021

**Published:** 2025-09-09

**Authors:** Asha S. George, Mary V. Kinney, Joy E. Lawn, Peter Waiswa

**Affiliations:** School of Public Health, Faculty of Community and Health Sciences, University of the Western Cape, Cape Town, Bellville, South Africa; Global Surgery Division, Department of Surgery, University of Cape Town, Cape Town, South Africa; London School of Hygiene & Tropical Medicine, Department of Infectious Disease Epidemiology and International Health, London, UK; School of Public Health, Makerere University College of Health Sciences, Kampala, Uganda

## Introduction

The landscape of global health financing has shifted profoundly in recent years – with redirection of donor aid to national security spending, economic shocks from the COVID-19 pandemic, and rising debt burdens – placing immense pressures on low- and middle-income countries (LMICs) to self-finance their health systems [1]. Sudden major donor cuts to global health amplify the need to transform health financing to more sustainable and equitable models that protect vulnerable populations, especially women, children, and adolescents [2,3]. Many LMICs, and notably those in sub-Saharan Africa, are off track or have slowed down in progressing towards the Sustainable Development Goals targets for ending preventable maternal, neonatal and child deaths by 2030. This requires urgent action and increased investment. Concurrently, global health initiatives (GHIs), including the Global Financing Facility (GFF), are also being reimagined to better support countries on their path towards Universal Health Coverage [4].

The GFF launched in 2015 with a compelling mission: to close financing gaps for women’s, children’s, and adolescents’ health through smarter, more sustainable investments [5]. Framed as a ‘country-led’ mechanism that could both mobilize and coordinate domestic and external financing, the GFF promised to move beyond traditional aid models by catalyzing investment cases tailored to each country’s priorities. A decade since its inception, there remains relatively little peer-reviewed research about how the GFF operates in practice, how its promises of country leadership and catalytic financing are realized, and what lessons can be drawn for the future of global health financing [4,6–9].

This editorial introduces a *Global Health Action* Special Issue comprising a series of papers based on multi-country, multi-disciplinary research, led primarily by African-based organizations and scholars, which aimed to fill the above mentioned evidence gap [10]. The articles explore GFF-related country policy content, processes, funding mechanisms, and country-level implementation from 2015 to 2022. In an era of declining foreign aid [3], bold models like the GFF are vital for mobilizing domestic resources and improving coordination. Yet their designs must be strengthened to meet their purpose and objectives. This editorial summarizes policy analyses of the initial billions of US dollars catalyzed through the GFF and reflects on the implications for today’s context.

## Overview of the Special Issue’s structure and collaborations

**Figure 1.**
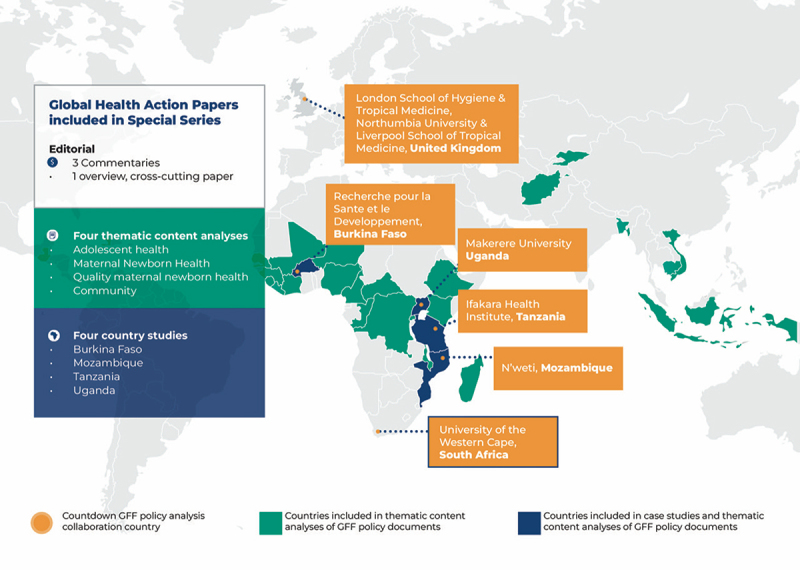
Mapping the Special Series papers- collaborators, and geographic reach of countries included in content analyses and/or case studies.

The Special Series, titled *Global Financing Facility for Women, Children, and Adolescents: Examining National Priorities, Processes, and Investments*, draws on qualitative interviews and analysis of GFF country planning documents – notably Investment Cases and GFF-linked World Bank Project Appraisal Documents (PADs) – during the first 7 years of operation (Table 1) [10]. Figure 1 maps the countries and documents included in the thematic content analyses, which totals 28 countries and 54 documents (24 investment cases and 30 PADs), as well as four country studies. In addition to the nine research papers, the Special Issue includes three commentaries: one from the GFF leadership [15]; one from the Partnership for Maternal, Newborn, and Child Health [16], and a third from African health systems experts [17]. The first paper in the Series describes and assesses the GFF’s multiple roles, maps their investments, and presents an overview of our cross-study findings [11]. It synthesizes results and lessons learned from four thematic content papers: adolescent health [18], maternal and newborn health (MNH) [19], MNH quality of care [20], and community engagement [21]) and the four country studies Burkina Faso [22], Mozambique [23], Tanzania [24], and Uganda [25].

**Table 1.** Understanding key terms related to GFF-related policy documents and linked funding.

Types of documents or funds	Description
Health Sector Plans	National strategic documents are developed and published by Ministries of Health to outline health goals, policies, and resource needs across the entire health system.
Investment Case	Country-led planning document co-developed and used by the government, GFF, and other partners to identify priority interventions for RMNCAH-N, published by the Ministry of Health. The Investment Case is supposed to align with national plans and be used to mobilize domestic and external resources. For this Special Series, Kinney et al (2025) identified that out of 28 countries: 18 had specific investment cases developed for GFF, four countries used their national health plans as Investment Cases, two countries had working documents, and four countries did not have investment cases [11].
World Bank Project Appraisal Document (PAD)	This document is a step in the World Bank’s project cycle that presents a proposed project before the World Bank provides funding [12].
GFF-linked PAD	These documents are PADs that fund a subset of priorities identified in the Investment Case and include the GFF grant. They are country-specific and present activities and components of the project, financed primarily by the International Development Association (IDA). Kinney et al (2025) identified 30 PADs across the 28 countries valuing at US$14.5 million overall: US$ 595 million from the GFF Multi-Donor Trust Fund (4%), US$ 3.5 billion from World Bank through IDA (23%), and US$ 10.05 billion from other funding, including government commitments (72%) [11].
International Development Association (IDA)	The part of the World Bank that supports low-income countries, offering grants and concessional loans with long repayment terms. IDA provides low- or no-interest loans (credits) and grants to low-income countries offering favorable terms but require repayment [13].
GFF Multi-Donor Trust Fund	A pooled financing mechanism housed at the World Bank that brings together contributions from multiple bilateral and philanthropic donors to support reproductive, maternal, newborn, child, and adolescent health and nutrition in low- and lower-middle-income countries. It is not a standalone source of funding, but rather a catalytic fund that seeks to align and amplify health investments through strategic co-financing and country-led planning [14].

In our assessment of GFF country policy documents, we examined the sequence whereby countries begin with broad health sector plans that set national priorities. From these, a more focused Investment Case to prioritize actions for women’s, children’s, and adolescents’ health is developed. Then, a World Bank Project Appraisal Document (PAD) is created to fund a subset of the stated priorities. The financing from the GFF Multi-Donor Trust is linked to World Bank projects, which also include financing from the International Development Association (IDA). Definitions of these policy documents and linked funding streams are provided in Table 1.

The Special Issue was done as part of Countdown to 2030 for Women’s, Children’s and Adolescents’ Health, which tracks and supports accountability for progress towards the Sustainable Development Goals [26]. In 2018, the Countdown Drivers Technical Working Group initiated a study to explore how adolescent health was addressed in the GFF country planning documents for the first 11 countries [27]. The study sparked more questions than answers, particularly regarding the processes by which priorities were set, decisions were made, and funds were disbursed. Subsequently, the Countdown to 2030’s Health Policy and Systems Working Group anchored at the School of Public Health at the University of the Western Cape [28], in collaboration with NEST360 Alliance [29], set out to further understand and explore GFF policy processes. From 2021 to 2024, a group of 17 people – researchers, policymakers, and civil society leaders – mostly from African organizations, formed the Countdown GFF Policy Analysis Collaboration (Figure 1). Country studies were led by in-country partners. Thematic content analyses were concurrently led by the University of the Western Cape, RESADE, the London School of Hygiene & Tropical Medicine, and Northumbria University.

The collaboration was shaped by a deliberate commitment to equitable partnership and South–South collaboration. The team co-designed the research through a participatory process, including in-person workshops (September 2021, October 2022, and March 2023) and regular, monthly online meetings to co-develop tools, discuss data collection and analysis processes, share preliminary results, and identify cross-cutting themes across studies. The collaboration intentionally engaged and supported emerging researchers, providing them with senior mentorship and robust peer learning support. A reading journal club on relevant health policy analysis articles was facilitated, and routine online meetings were convened by all participating organizations on a rotational basis.

The work also went beyond academic inquiry, by regularly feeding back preliminary and final results with the GFF Secretariat and Ministries of Health. The sharing of preliminary results was critical given that little was known about the GFF model when we started. Initial analysis focused on GFF country planning documents (investment cases and the GFF-linked World Bank project documents) because this was all that was publicly available on the GFF webpage when the research started. Iterative debriefing sessions with the GFF enabled the team to better understand the nuances and challenges involved, appreciate the differences in interpreting evidence and the clarifications needed, especially as the reported goal posts for assessment kept changing. Preliminary findings were also shared at international conferences facilitating broader discussion and sense making. This iterative exchange created opportunities for real-time reflection and adaptation, both within countries and at the global level.

## What we learned about the GFF

The articles in this Series highlight both the promises and challenges of the GFF model based on our learnings from their initial years [11]. The first paper provides cross-cutting findings of what we learned – that while the GFF in its first 7 years mobilized US$ 14.5 billion across 28 countries, its implementation varied widely, shaped by technocratic processes, political negotiations, and country-specific dynamics [11]. Here, we reflect more broadly on what was learned and the approaches that were taken.

First, the GFF promotes itself as a mechanism that supports countries to identify priorities, mobilize resources, convene stakeholders, and enable continuous learning [30]. Our Series shows that the GFF was indeed a mechanism to support countries in these activities and helped to elevate reproductive, maternal, newborn, child and adolescent health (RMNCAH) in national agendas and mobilize resources by leveraging World Bank financing. At the same time, the realities of its policy process and implementation expose persistent tensions around power, process, and purpose.

Our study found that the GFF operates within a highly political and power-laden ecosystem, shaped significantly by its location within the World Bank [11]. Unlike other GHIs that provide standalone grants to governments and implementing partners, GFF financial support is embedded within World Bank lending projects. Countries must borrow – often through International Development Association (IDA) credits – to unlock GFF grants. This coupling of loans and grants effectively means that participation in the GFF is contingent on willingness (and capacity) to commit domestic resources through debt from a development bank that wields influence with national governments because of its lending across all sectors, not just health. Even if the debt is on heavily discounted terms, for many GFF countries already under fiscal strain, this structure poses specific challenges to long-term accountability and sustainability.

Second, GFF policy processes, while overlaid with evidence and technical guidance, are nonetheless ultimately negotiated by multiple actors that shape its expression at country level. Although the GFF emphasizes country leadership, it’s technocratic aspects limited understanding and engagement by other actors. Investment Cases are framed as nationally determined priorities and were reported to be developed through inclusive processes [11,31]. Yet stakeholders also reported that key decisions – such as indicator selection and intervention focus – were strongly influenced by a smaller group of actors. Civil society actors, meanwhile, were largely excluded in early GFF planning cycles and especially the policy processes linked to the PADs [11]. Their engagement, when it initially occurred, was often ad hoc and under-resourced.

Despite these dynamics, country actors actively engaged with the GFF, often strategically. They navigated donor expectations, leveraged the GFF to align fragmented donor efforts, and used the process to advocate for longstanding national priorities. Their experiences highlight the complex, negotiated nature of country ownership in global health financing. However, even with the development of consensus-based Investment Cases, this did not necessarily always translate into impactful investments or implementation, as discussed below.

A third insight, drawn from our thematic content analyses, was the disconnect at times between the ambitions laid out in the country Investment Cases and the operational details of corresponding GFF-linked World Bank PADs [11]. While Investment Cases are designed as comprehensive planning documents for RMNCAH, PADs represent a specific World Bank project linked to GFF multi-donor trust grants. Because PADs are governed by World Bank procedures and budget ceilings, their scope is narrower, leading at times to the exclusion of critical health indicators or population groups, included in the Investment Cases but not the PADs. The final investments often prioritized system-level reforms, such as results-based financing, that align with broader World Bank agendas on health system strengthening. The limited set of indicators in PADs reflects not only operational constraints but also deeper issues of measurement and accountability. These key indicators maybe included in further restructured project agreements or mid-term reviews that are not publically available. This weakens efforts to ensure that commitments are transparently monitored, resourced, and acted upon.

The GFF-linked World Bank projects are a key funding stream, but they represent only part of the resources intended to support Investment Cases. A central GFF goal is to mobilize additional funding and coordinate with donors and governments, and the GFF has worked with countries and partners to map RMNCAH resource allocations by donors and governments [32]. However, their methodology for this resource mapping is not explained on their website nor published in peer-review literature [32]. Likewise, their impact as a catalytic fund for resource mobilization, beyond the World Bank credit, was challenging to assess and has not been demonstrated by others [31,33].

Finally, and one of the more encouraging findings from our work, is the degree to which the GFF has been willing to evolve overtime. Specific to our collaboration, GFF stakeholders engaged meaningfully with the research team. The level of openness to critique and collaboration is not common among global health institutions and reflects a commendable willingness to learn and adapt.

In addition, during our research, the GFF enhanced its monitoring and evaluation frameworks, introduced new learning tools, and updated key guidance documents and web resources. The creation of a results team and a more user-friendly knowledge platform (https://data.gffportal.org/) signaled a shift toward better knowledge management and learning. Critically, the GFF also facilitated its own independent evaluation in 2024, with similar findings to what is documented in this Series [31,33]. Nevertheless, institutional evolution must go together with deeper structural reforms – particularly around financing models and their governance.

## Priorities going forward

Over the past decade, the GFF has brought welcome attention and resources to RMNCAH. The goal must now be to transform that momentum into systemic, sustainable change, given diminishing development assistance combined with stiffer competition with regard to attribution. Drawing from the findings in this series, we propose three priorities for reform:

### Money: shift from gap-filling loans to enabling national financing transformation

While the GFF’s model of linking its financing to World Bank lending has enabled countries to access significantly more funding than traditional grants alone could provide, this strategy comes with trade-offs. In countries with already limited fiscal space, reliance on concessional loans can exacerbate debt burdens and constrain future public spending.

Going forward, the GFF must more strongly advocate for grant-based support and invest in domestic financing capabilities, including tax reform, budget execution, and public financial management. The goal should not be to fill funding gaps temporarily, but to also further support countries build resilient, self-sustaining health financing systems for the long-term. To advance this agenda, the GFF must rebalance its financing strategy toward long-term system resilience. Loan-linked financing often incentivizes short-term, project-based interventions – reinforced by disbursement-linked indicators that can divert focus from the institutional reforms needed for sustained impact. The GFF should also prioritize tracking progress on expanding fiscal space for health and strengthening essential public health functions and national health financing units. The development of ‘resilience indicators’ [34] may help measure countries’ growing capacity to generate, manage, and sustain their own health financing – reinforcing a shift from temporary gains to deeper structural transformation.

### People, platforms, and power: invest in inclusive, country-ownership and coordination

Coordination is one of GFF’s core functions with the aim to strengthen national ownership through inclusive, nationally led platforms that align all partners behind a single investment case and national plan [30]. The functionality, inclusiveness, and influence of these country platforms vary widely, especially for inclusion of civil society and private sector. The GFF must do more to strengthen and sustain these platforms. Inclusion must be meaningful, not symbolic – and this will require ongoing resourcing and capacity strengthening. Likewise, countries need to be more proactive in enabling and supporting GFF policy processes, without depending on them. The future of RMNCAH and its financing ultimately lies with country actors; therefore, they need to be at the helm and supported in doing so.

### Count for accountability: strengthen measurement systems, transparency and multi-stakeholder learning

Even with the impressive GFF data portal and long indicator lists in the Investment Cases, too often PADs failed to include critical indicators – such as those for quality of maternal and newborn care including respectful care and stillbirth, which also risks missing impact. Continued investments in strengthening national health information systems as the backbone for routine and rapid monitoring and accountability are critical. Emphasis on collecting data beyond service coverage to include service quality and equity is key. Building on such data, independent monitoring mechanisms with networked local, regional and global stakeholders, need continued support with mandates and resources to assess whether GFF funding is truly catalytic, that is whether it mobilizes new and additional resources, aligns partners, and accelerates impact. Transparency in funding flows, project design, and indicator selection to support both accountability and learning, particularly if health system strengthening processes are to have impacts on RMNCAH outcomes, including outstanding mortality targets must be prioritized. Without meaningful measurement and accountability, health system strengthening efforts may fall short of delivering the RMNCAH outcomes that matter most – including progress on outstanding mortality targets.

## Conclusion

The Global Financing Facility is an important – and still evolving – ambitious experiment in how development partners and LMIC governments can support health priorities in the highest-burden settings. It offers lessons for RMNCAH financing and for the design of future global health initiatives. As this Series shows, the GFF’s strength lies in its ambition and its willingness to adapt. But ambition must be matched by structural alignment. That means addressing the power imbalances that make the difference between country led versus country owned plans and implementation, strengthening transparent country platforms, and supporting financing strategies to sustain health systems for true impact. As global health enters an era of reduced donor appetite and heightened demand for results, the GFF’s trajectory will shape not only maternal and child health outcomes but also the future of sustainable health financing in the Global South. This is a critical moment for bold recalibration and renewed commitment.

As the GFF enters its new strategic plan, the evidence presented here provides a foundation for reform. If the GFF is to deliver on its promise of sustainable, equitable health financing, it must more openly embrace the realities of politics, power, and partnerships and enable strengthened mechanisms of accountability, participation, and transparency.

This Special Series is a call to action. Not just for the GFF, but for those in the RMNCAH community who have a stake in reforming the future of global health financing to ensure impact for the world’s most vulnerable populations.
